# Supplementing Soy-Based Diet with Creatine in Rats: Implications for Cardiac Cell Signaling and Response to Doxorubicin

**DOI:** 10.3390/nu14030583

**Published:** 2022-01-28

**Authors:** Laurence Kay, Lucia Potenza, Isabelle Hininger-Favier, Hubert Roth, Stéphane Attia, Cindy Tellier, Christian Zuppinger, Cinzia Calcabrini, Piero Sestili, Theo Wallimann, Uwe Schlattner, Malgorzata Tokarska-Schlattner

**Affiliations:** 1 University Grenoble Alpes and Inserm U1055, Laboratory of Fundamental and Applied Bioenergetics (LBFA) and SFR Environmental and Systems Biology (BEeSy), 38059 Grenoble, France; Laurence.Kay@univ-grenoble-alpes.fr (L.K.); isabelle.hininger@univ-grenoble-alpes.fr (I.H.-F.); hubert.roth@laposte.net (H.R.); stephane.attia@univ-grenoble-alpes.fr (S.A.); cindy.tellier@univ-grenoble-alpes.fr (C.T.); 2 Dipartimento di Scienze Biomolecolari, Università Degli Studi di Urbino “Carlo Bo”, 61029 Urbino, Italy; lucia.potenza@uniurb.it (L.P.); cinzia.calcabrini@uniurb.it (C.C.); piero.sestili@uniurb.it (P.S.); 3 Inselspital, Cardiology Department, Bern University Hospital, 3010 Bern, Switzerland; christian.zuppinger@dbmr.unibe.ch; 4 Department of Biology, ETH-Zurich, Emeritus, 8962 Bergdietikon, Switzerland; theo.wallimann@cell.biol.ethz.ch

**Keywords:** adenosine 5′-monopnophosphate-activated protein kinase, anthracyclines, creatine supplementation, cardiac signaling, cardiotoxicity, doxorubicin, soy, vegetarian/vegan diet

## Abstract

Nutritional habits can have a significant impact on cardiovascular health and disease. This may also apply to cardiotoxicity caused as a frequent side effect of chemotherapeutic drugs, such as doxorubicin (DXR). The aim of this work was to analyze if diet, in particular creatine (Cr) supplementation, can modulate cardiac biochemical (energy status, oxidative damage and antioxidant capacity, DNA integrity, cell signaling) and functional parameters at baseline and upon DXR treatment. Here, male Wistar rats were fed for 4 weeks with either standard rodent diet (NORMAL), soy-based diet (SOY), or Cr-supplemented soy-based diet (SOY + Cr). Hearts were either freeze-clamped in situ or following ex vivo Langendorff perfusion without or with 25 μM DXR and after recording cardiac function. The diets had distinct cardiac effects. Soy-based diet (SOY vs. NORMAL) did not alter cardiac performance but increased phosphorylation of acetyl-CoA carboxylase (ACC), indicating activation of rather pro-catabolic AMP-activated protein kinase (AMPK) signaling, consistent with increased ADP/ATP ratios and lower lipid peroxidation. Creatine addition to the soy-based diet (SOY + Cr vs. SOY) slightly increased left ventricular developed pressure (LVDP) and contractility dp/dt, as measured at baseline in perfused heart, and resulted in activation of the rather pro-anabolic protein kinases Akt and ERK. Challenging perfused heart with DXR, as analyzed across all nutritional regimens, deteriorated most cardiac functional parameters and also altered activation of the AMPK, ERK, and Akt signaling pathways. Despite partial reprogramming of cell signaling and metabolism in the rat heart, diet did not modify the functional response to supraclinical DXR concentrations in the used acute cardiotoxicity model. However, the long-term effect of these diets on cardiac sensitivity to chronic and clinically relevant DXR doses remains to be established.

## 1. Introduction

Nutritional habits are increasingly recognized for their impact on cardiovascular health and disease, including prevention of cancer relapse or different comorbidities [1]. This is of particular interest for acute or chronic cardiotoxicity caused by anticancer chemotherapeutics, in particular anthracyclines, such as doxorubicin (DXR; reviewed in [2,3,4]), for which efficient preventive or therapeutic strategies are still lacking. Currently, cardiotoxic side effects are minimized by limiting total drug dose, slow administration by infusion rather than bolus injection, liposomal drug encapsulation, or co-administration of the iron chelator dexrazoxane, a protective adjuvant only approved in the USA [5,6,7]. More recent recommendations to patients have started to emphasize lifestyle changes, including physical activity (exercise) and nutritional habits [1,8,9,10,11,12].

Creatine (Cr) is one of the most popular dietary supplements [13]. Humans synthesize only about 50% of their daily Cr requirement in the kidney, pancreas, and liver [14] and certain brain cells [15]. The remainder has to be taken up from Cr-containing non-vegan nutrition, especially fish and meat [16]. Cr then enters cells, such as cardiomyocytes, via the plasma membrane Cr transporter. Cr constitutes a cellular energy precursor, transformed within the cell into the “energy-rich” phosphocreatine (PCr). Cr and PCr, together with isoforms of creatine kinase (CK), confer bioenergetic advantages by providing an efficient energy buffer and transfer system for cells and tissues with high and fluctuating energy requirements, such as the heart [17]. Supplementation with chemically pure Cr shows protective effects in different pathologies, such as cardiac ischemia and reperfusion injury ([18]; for a review, see [19]) or neurodegenerative and muscular disorders (reviewed in [16]). Some protective effects were also observed with DXR-induced injury, including reduced cardiac damage in DXR-treated animals [20], improved viability of DXR-treated cultured cardiomyocytes and H9c2 cells [21,22], and protection against DXR-induced RNA damage in non-cardiac cells [23]. CK overexpression in a murine model of DXR cardiotoxicity improved myocardial energetics, contractile dysfunction, and survival [24]. Beyond the bioenergetic functions of Cr and PCr, pleiotropic anti-oxidative and anti-apoptotic effects were reported, including reduced mitochondrial ROS, increased oxidative stress defense, and inhibition of mitochondrial permeability transition (reviewed by [16,25]). Cr and PCr also interact with anionic membrane phospholipids, increasing phospholipid packing and thus stabilizing and protecting biomembranes [26].

Cr supplementation is of particular interest in combination with vegetarian/vegan diets that naturally do not contain Cr. Using soy-derived products (soy meal or soy protein isolate) to replace meat- and fish-derived compounds as the main protein source yields a Cr-free diet, which can serve as an experimental control for Cr supplementation. However, soy-based products have been reported to also mediate protective metabolic effects on their own, including on the cardiovascular system [27,28,29,30]. Some of the bioactive compounds in soy-derived products are supposed to be isoflavones (genistein, daidzein, and equol) that may act as phytoestrogens [28].

Here, we investigated whether a soy-based vegan chow (SOY) as compared to a standard rodent chow (NORMAL), and a Cr-supplemented soy chow (SOY + CR) as compared to SOY, can affect cardiac function, biochemistry, and cell signaling in general, and the cardiac response to DXR in particular. In our case, NORMAL contained soy meal and fish hydrolysate as protein sources, with the latter containing a variable amount of naturally occurring Cr. SOY was entirely vegan, with soy products as the exclusive protein source and entirely lacking Cr, while SOY + CR was supplemented with 2% (*w*/*w*) chemically pure synthetic Cr monohydrate. Our data show that one month of being fed the differential diet has modest but significant functional and biochemical effects on the rat heart, in particular on specific cell signaling pathways. Some of them are potentially relevant for cardiac health and the response to DXR, but they did not confer functional improvement in the perfused heart model of acute DXR cardiotoxicity.

## 2. Materials and Methods

### 2.1. Materials

Doxorubicin hydrochloride (DXR) was purchased from Sigma (Saint Louis, MO, USA) or Selleck Chemicals (Houston, TX, USA). A stock solution (10 mM) was prepared in water and kept frozen until use. Further dilutions were prepared in Krebs-Henseleit buffer [31] just before heart perfusion. Protease inhibitor cocktail tablets were obtained from Roche (Mannheim, Germany) and phosphatase inhibitor cocktail was obtained from Pierce (Rockford, IL, USA). Creatine (creatine monohydrate, Creapure^®^) was a gift from AlzChem Trostberg GmbH (Trostberg, Germany).

### 2.2. Animals

**Figure 1:**
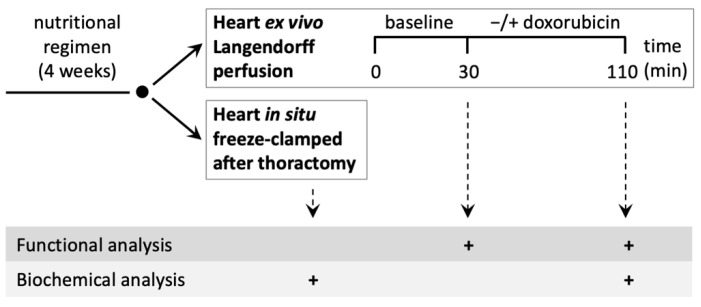
Scheme of the experimental procedure (for details, see Material and Methods).

All procedures involving animals were approved by the Grenoble Ethics Committee for Animal Experimentation (15_LBFA-U884-HD-01). Male Wistar rats initially fed a standard chow for young rats (A03 reference U8200, Safe, Augy, France; 3237 kcal/kg) were then differentially fed for 4 weeks starting from 2 months of age. One group of animals continued to receive the standard chow for adult rats (NORMAL; A04 reference U8220, Safe, Augy, France; 2791 kcal/kg) containing 4% (*w*/*w*) fish hydrolysate and 8% (*w*/*w*) soy meal. The second group was fed a Cr-free soy-based chow (SOY; modified A04 reference U8220 version 149, Safe, Augy, France; 2711 kcal/kg) where fish hydrolysate was replaced by the same percentage of soy isolate. The third group was fed the latter chow supplemented with 2% (*w*/*w*) creatine (SOY + Cr, modified A04 reference U8220 version 150, Safe, Augy, France; 2711 kcal/kg). Diets were purchased from Safe, Augy, France). After 4 weeks of the differential diets, animals were anaesthetized with sodium pentobarbital (50 mg/kg i.p.), and hearts were freeze-clamped in situ (immediately after thoracotomy of respirator-ventilated animals) or following ex vivo Langendorff perfusion with or without 25 μM DXR (see the experimental scheme in Figure 1). Frozen hearts were stored at −80 °C.

### 2.3. Rat Heart Perfusion

Perfusion experiments were essentially performed according to the protocol described earlier [31,32,33,34]. Briefly, rats were anaesthetized with sodium pentobarbital (50 mg/kg i.p.) and heparinized (1500 IU/kg i.v.). Hearts were quickly removed and perfused at constant pressure in a non-circulating Langendorff apparatus with Krebs-Henseleit buffer, first for 30 min for stabilization (baseline) with Krebs-Henseleit buffer alone, and then for 80 min either with Krebs-Henseleit buffer without (control) or with 25 μM DXR. The DXR concentration was chosen on the basis of our previous studies [31,32,33,34]. During perfusion, systolic pressure, end diastolic pressure, dp/dt and –dp/dt, and heart rate were recorded every 10 min.

### 2.4. Metabolites, Oxidative Damage/Antioxidant Status, and DNA Integrity

Protein-free extracts were obtained by perchloric acid precipitation, and metabolites were quantified using HPLC (AMP, ADP, ATP) or a spectrophotometric assay (Cr and PCr) as described earlier [32,34]. Markers of oxidative damage and antioxidant status were quantified in heart extracts prepared as described earlier [35]. Reduced thiol (SH) groups were assayed according to [36]. N-acetyl cysteine (NAC) in the range of 0.125 to 1 mM (prepared from a 100 mM stock solution) was used for calibration. Standards and heart extracts were diluted in 50 mM phosphate buffer, 1 mM EDTA, pH 8, and 2.5 mM 5,5′-dithio-bis-(2-nitrobenzoic acid) (DTNB), and subsequently the absorbance was measured at 412 nm. Antioxidant status was evaluated using the ferric reducing ability power (FRAP) assay [37]. Plasma thiobarbituric acid reactive substance (TBARS) concentrations were assessed as described [38]. Total genomic DNA was isolated with the QIAamp DNA mini kit (Qiagen) according to the manufacturer’s instructions. The final concentration and quality of DNA were estimated both spectrophotometrically (DU-640; Beckman Instruments, Milan, Italy) at 260 nm, and by agarose gel electrophoresis. Nuclear and mitochondrial DNA damage were evaluated using a two-step strategy based on a long PCR and real-time PCR as described in detail elsewhere [34].

### 2.5. Immunoblotting

SDS-PAGE separation of heart homogenates (40–50 µg) and immunoblotting were performed according to standard procedures [34]. The transfer quality and equality of loading were checked by Ponceau staining. The blots were developed with chemiluminescence reagent (ECL Prime, GE Healthcare) using a CCD camera (ImageQuant LAS 4000, GE Healthcare). The quantification of signals was conducted using ImageQuantTL software (GE Healthcare). Tubulin or total protein were used for normalization for the phosphorylated proteins (probed on different membranes). The following primary antibodies obtained from Cell Signaling (Beverly, MA) were used: anti-AMPKα (Cat# 2532, RRID:AB_330331), anti-P(Thr172)AMPKα Cat# 2535, RRID:AB_331250), anti-ACC (Cat# 3662, RRID:AB_2219400), anti-P(Ser79)ACC (Cat# 3661, RRID:AB_330337), anti-Akt (Cat# 4691, RRID:AB_915783), anti-P(Ser473)Akt (Cat# 4060, RRID:AB_2315049), anti-P(Thr308)Akt (Cat# 13038, RRID:AB_2629447), anti-ERK-1/2 (Cat# 4695, RRID:AB_390779), anti-P(Thr202/Tyr204)ERK-1/2 (Cat# 4370, RRID:AB_2315112), anti-tubulin (Cat# 2128, RRID:AB_823664), anti-P(Ser/Thr)Akt Substrate Motif RXXS/T (Cat# 9614, RRID:AB_331810).

### 2.6. Data Analysis

Results are expressed as means ± SEM, if not stated otherwise. Depending on the experimental design, statistical analysis was performed using one- or two-way ANOVA (Sigma Plot; Systat Software, San Jose, CA, USA) or linear regression with dummy variables and robust standard errors (Stata 13; Stata Corp., College Station, TX, USA) to deal with the heterogeneity of variance. When appropriate, these were followed by the Student–Newman–Keuls or Bonferroni test, respectively, for pairwise comparisons. The one-way ANOVA P-values are not reported; the results of pairwise comparisons are reported in the case of significant one-way ANOVA P-values. For two factorial analysis, we report significance values for the effects of diet (P_Diet_, independent of DXR), DXR (P_DXR_, independent of diet), and the interaction of both diet and DXR treatment (P_Diet*DXR;_ indicating if the effect of one factor depends on the level of the second factor), and the results of pairwise comparisons (*p*). The P or *p* values are given in the graphs with 3 decimal places (and in bold characters) if significant, and with 2 decimal places if not. A value of P or *p* < 0.05 (for interaction P < 0.1) was considered statistically significant.

## 3. Results

The effects of the three nutritional regimens, standard rodent chow (NORMAL), soy-based diet (SOY), and Cr-supplemented soy-based diet (SOY + Cr), on the heart under control and DXR-challenged conditions were studied in a rat model (Figure 1). After one month of the differential diet, there was no significant difference in animal body weight (NORMAL 366 ± 6 g (*n* = 36), SOY 387 ± 12 g (*n* = 23), SOY + Cr 370 ± 11 g (*n* = 21)). Biochemical parameters were determined both in hearts freeze-clamped in situ immediately after thoracotomy, and after ex vivo Langendorff perfusion, consisting of a 30 min stabilization period followed by 80 min of perfusion without (control group) or with 25 μM DXR (DXR group). Functional parameters were measured ex vivo during Langendorff perfusion.

### 3.1. Heart Function

**Figure 2:**
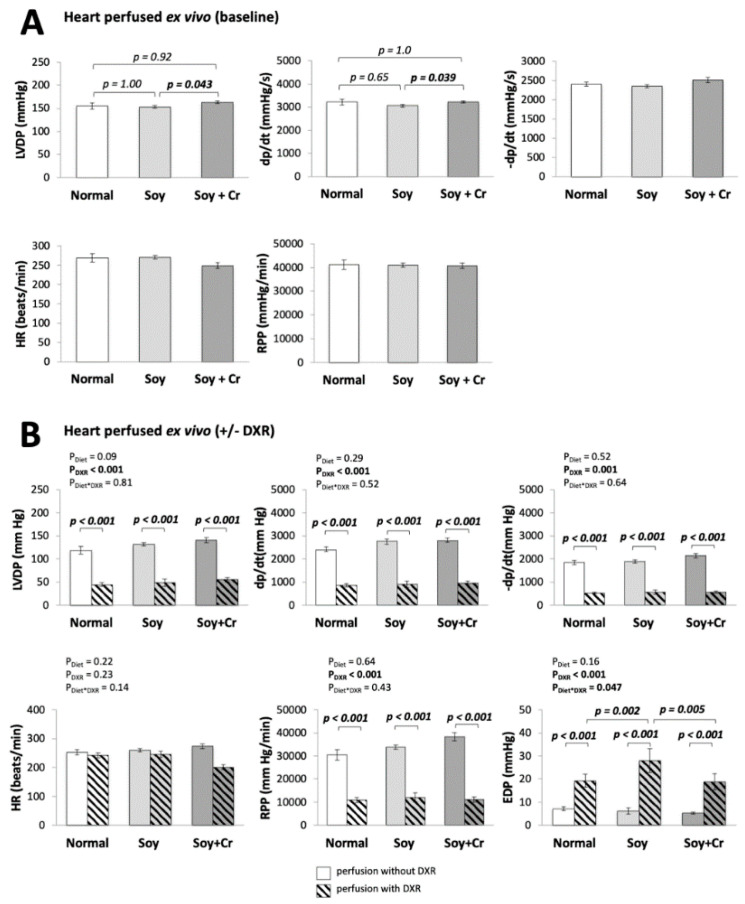
Heart function: effect of diet and DXR. Hemodynamic parameters: left ventricular developed pressure (LVDP), end-diastolic pressure (EDP), dp/dt, −dp/dt, heart rate (HR), and rate pressure product (RPP) measured in Langendorff perfused hearts after 30 min of stabilization (**A**) or after 30 min of stabilization followed by an additional 80 min of perfusion (**B**) without or with 25 μM DXR (empty or hatched bars, respectively). EDP values are given only in (**B**), as during the stabilization period shown in (**A**), EDP was adjusted to 5 mm Hg and thereafter the volume of the balloon rest unchanged. Statistical analysis with linear regression followed by the Bonferroni test for pairwise comparisons. For −dp/dt, HR, RPP in (**A**), and HR in (**B**), there are no statistically significant differences between groups. Mean ± SEM, *n* = 11–28 (**A**), *n* = 4–14 (**B**).

Cardiac function was first determined in perfused hearts at baseline (after 30 min of stabilization, Figure 2A), and then after 80 min of subsequent perfusion without or with DXR (Figure 2B). Data at baseline are considered to reflect the in vivo situation. The Cr-supplemented diet (SOY + Cr vs. SOY, Figure 2A) affected cardiac function, with a slight increase in the cardiac developed pressure LVDP (*p* = 0.043) and contractility dp/dt (*p* = 0.039). There was no significant effect of soy diet (SOY vs. NORMAL). DXR perfusion impaired almost all cardiac functional parameters (P_DXR_ < 0.001, Figure 2B) except heart rate, with a time-course (Figure S1) consistent with previous studies [31,32,33,34]. A statistically significant interaction between diet and DXR was only seen for diastolic pressure at the end of perfusion (EDP; P_Diet*DXR_ = 0.047, Figure 2B), increasing more in the group fed the Cr-free soy chow (SOY vs. NORMAL, *p* = 0.002; SOY vs. SOY + Cr, *p* = 0.005).

### 3.2. Creatine and Adenylate Levels

**Figure 3:**
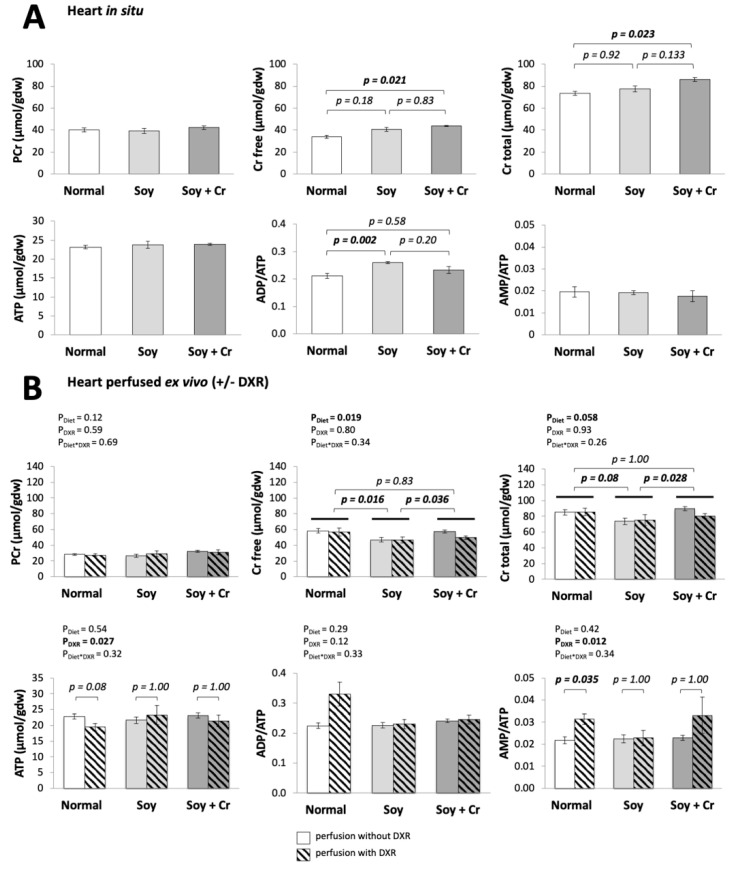
Energy metabolite levels: effect of diet and DXR. PCr, free Cr, total Cr, ATP, ADP/ATP, and AMP/ATP ratios in hearts freeze-clamped in situ immediately after thoracotomy (**A**) or following ex vivo Langendorff perfusion (**B**) without or with 25 μM DXR (empty or hatched bars, respectively). Statistical analysis with linear regression followed by the Bonferroni test for pairwise comparisons. For PCr, ATP, and AMP/ATP in (**A**) and PCr and ADP/ATP in (**B**), there are no statistically significant differences between groups. Mean ± SEM, *n* = 3–6 (**A**), *n* = 4–16 (**B**).

Cellular Cr availability and energy state were studied by determination of the Cr and adenylates in heart in situ and after ex vivo perfusion. A deteriorated energy state is often observed in DXR cardiotoxicity [39]. The diets affected the free and total Cr content in situ (Figure 3A) and also after ex vivo perfusion (P_Diet_ = 0.019 and 0.058, respectively, Figure 3B). As expected, 4 weeks of oral Cr supplementation (SOY + Cr vs. SOY) increased free and total Cr (*p* = 0.036 and *p* = 0.028, respectively, in ex vivo perfused hearts).

Interestingly, standard chow-fed animals also showed increased Cr in comparison to SOY (NORMAL vs. SOY), but the effect was seen only in the group used for ex vivo perfusion (*p* = 0.016 for free Cr and strong tendency *p* = 0.08 for total Cr), possibly due to a higher Cr content in the batch of NORMAL chow used here. Cardiac adenylate levels and ADP/ATP and AMP/ATP ratios remained largely unchanged between the diets (Figure 3A,B, lower rows), except for the soy chow, where the ADP/ATP ratio increased in hearts clamped in situ (SOY vs. NORMAL, *p* = 0.002). DXR perfusion across all nutritional regimens affected the ATP content and increased AMP/ATP ratios (P_DXR_ = 0.027 and 0.012, respectively, Figure 3B). No statistically significant interference was found between diet and DXR (Figure 3B).

### 3.3. Cell Signaling Pathways

**Figure 4:**
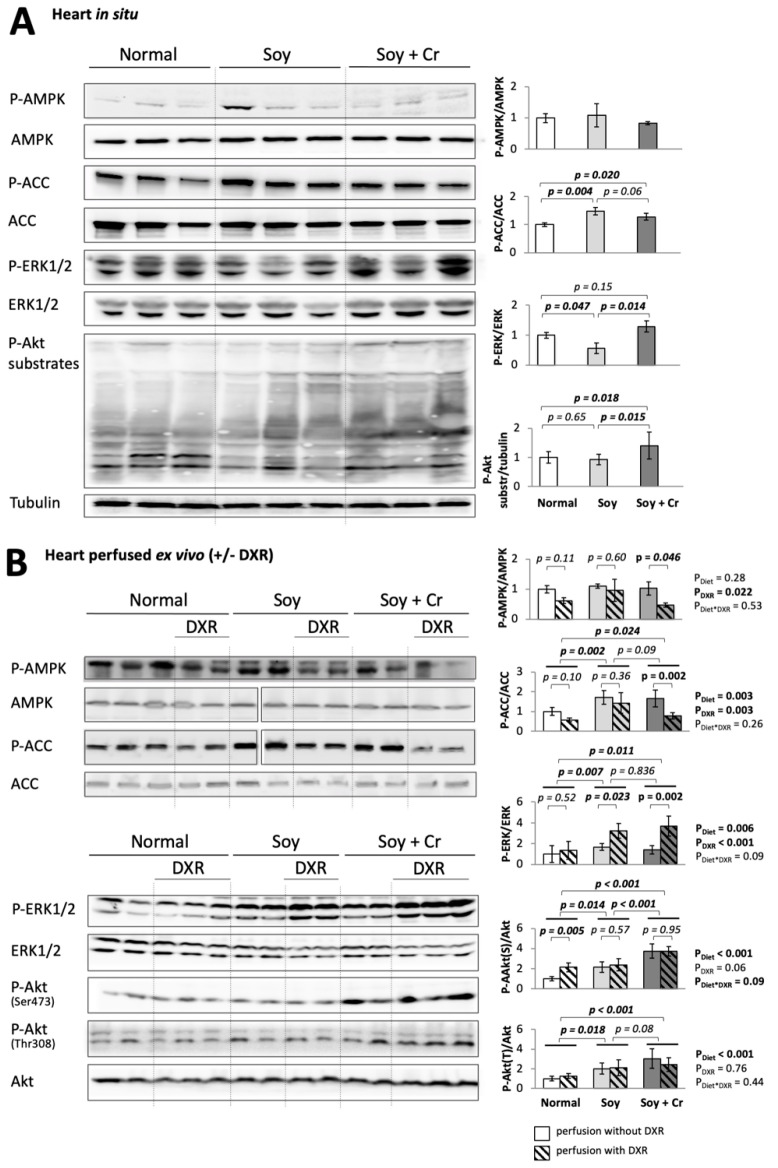
Activation of signaling pathways: effect of diet and DXR. Activation of cardiac pro-survival signaling pathways (AMPK, Akt, ERK) probed by immunoblot in total homogenates of hearts freeze-clamped in situ immediately after thoracotomy (**A**) or following ex vivo Langendorff perfusion (**B**) without or with 25 μM DXR. The P-ACC/ACC ratio and phosphorylated Akt substrates are a readout for the activation of the AMPK and Akt pathways, respectively. The quantification of the bands is given in the right panel; in (**B**), empty or hatched bars correspond to perfusion without or with 25 μM DXR, respectively. For each protein, all signals originate from the same blot. Total protein or tubulin signals were used for normalization. Statistical analysis with one-way (**A**) or two-way (**B**) ANOVA followed by the Student–Newman–Keuls test for pairwise comparisons. For P-AMPK/AMPK in (**A**), there is no statistically significant difference between groups. Mean ± SD, *n* = 3–5.

Nutrition can lead to sustained alterations in cell signaling, and this can also occur with DXR treatment as we have shown earlier [31,32,33,34]. We therefore analyzed the activation of specific key signaling pathways involved in stress and pro-survival responses: AMP-activated protein kinase (AMPK; determined by phosphorylation of AMPK itself and its substrate acetyl-CoA carboxylase, ACC), extracellular signal-regulated kinase (ERK, determined by ERK phosphorylation), and Akt (determined by either Akt phosphorylation or global phosphorylation of Akt substrates) in heart in situ (Figure 4A) and after ex vivo perfusion (Figure 4B). Our experiments revealed a differential cardiac activation pattern of these signaling pathways, dependent on the diet (Figure 4A,B). The soy-based diet (SOY vs. NORMAL) almost doubled ACC phosphorylation (*p* = 0.004 in situ; *p* = 0.002 after ex vivo perfusion), consistent with the above-described increase in the ADP/ATP ratio. Changes in P-AMPK were similar but weaker and did not reach significance. The addition of Cr to the soy-based diet (SOY + Cr vs. SOY) led to no further change in P-ACC but activated ERK (*p* = 0.014 in situ) and Akt (*p* = 0.015 in situ; *p* < 0.001 after ex vivo perfusion at Ser473, and a tendency with *p* = 0.08 at Thr308). Perfusion with DXR changed the phosphorylation of AMPK, ACC, ERK, and Akt at Ser473 (P_DXR_ = 0.022, P_DXR_ = 0.003, P_DXR_ < 0.001, strong tendency with P_DXR_ = 0.06, respectively, Figure 4B). This confirmed our previous observations in animals fed a NORMAL diet [31,34], namely a DXR-induced inactivation of AMPK signaling with a decrease of P-AMPK and P-ACC (tendencies of *p* = 0.11 and 0.10, respectively), together with an activation of Akt (*p* = 0.005 at Ser473) as one factor potentially involved in AMPK inactivation [34]. For phosphorylation of Akt at Ser473, the interaction between diet and DXR was significant (P_Diet*DXR_ = 0.09, Figure 4B), with an increase only observed in the NORMAL group.

### 3.4. Oxidative Damage, Antioxidant Status, and DNA Integrity

**Figure 5:**
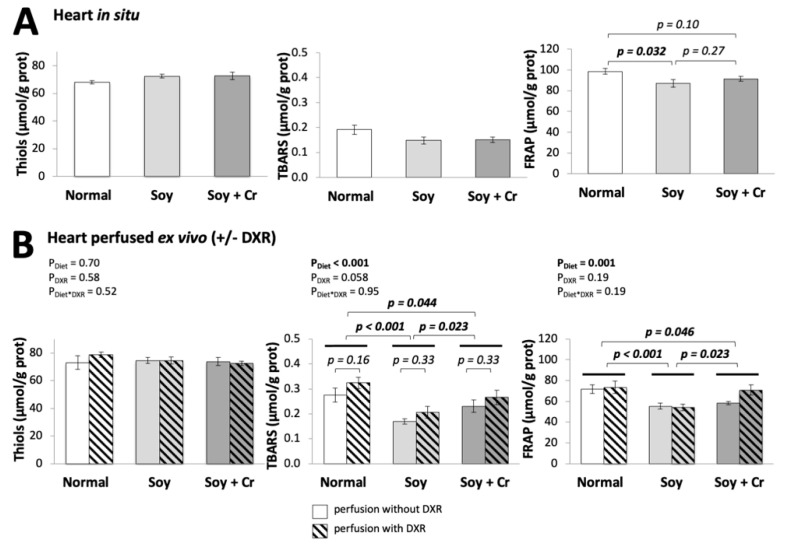
Oxidative/antioxidant status: effect of diet and DXR. Reduced thiols, peroxidized lipids (TBARS), and total antioxidant power (FRAP) measured in hearts freeze-clamped in situ immediately after thoracotomy (**A**) or following ex vivo Langendorff perfusion (**B**) without or with 25 μM DXR (empty or hatched bars, respectively). Statistical analysis with one-way (**A**) or two-way (**B**) ANOVA followed by the Student–Newman–Keuls test for pairwise comparisons. For thiols, TBARS in (**A**) and thiols in (**B**), there are no statistically significant differences between groups. Mean ± SEM, *n* = 5–6 (**A**), *n* = 4–7 (**B**).

**Figure 6:**
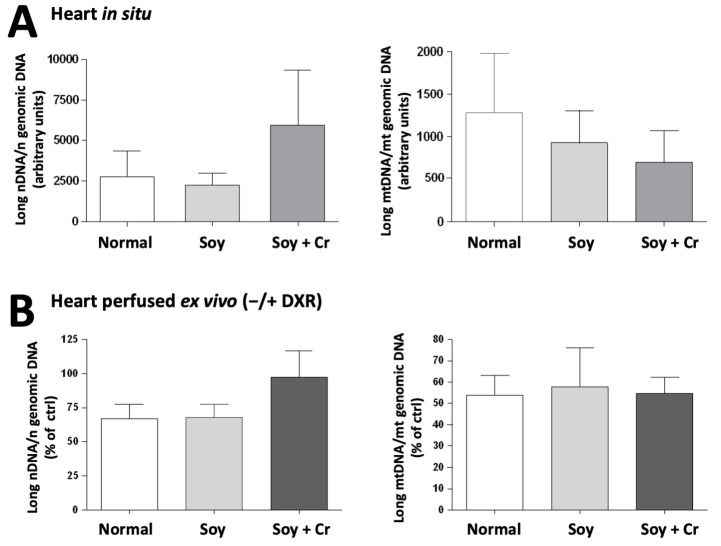
DNA integrity: effect of diet and DXR. Integrity of nuclear and mitochondrial DNA measured in hearts freeze-clamped in situ immediately after thoracotomy (**A**) or following ex vivo Langendorff perfusion (**B**). The bars in (**B**) represent the ratio of amplification values of DXR-perfused hearts to controls (perfusion without DXR) and are expressed as a percent. Statistical analysis with one-way ANOVA. For any parameter, there is no statistically significant difference between groups. Mean ± SD, *n* = 4–5.

Diet and DXR can affect the cellular oxidative/antioxidant balance. As a readout, we determined peroxidized lipids (TBA reactive substances, TBARS) and antioxidant status (reduced thiols; ferric reducing antioxidant power, FRAP), along with the integrity of mtDNA and nDNA in hearts in situ (Figure 5A and Figure 6A) and after ex vivo perfusion (Figure 5B and Figure 6B). In Situ, the soy-based diet diminished FRAP (SOY vs. NORMAL; *p* = 0.032, Figure 5A). In the ex vivo perfused heart, the diets affected both TBARS and FRAP (P_Diet_ < 0.001 and 0.001, respectively, Figure 5B). Again, the soy-based diet reduced both parameters (SOY vs. NORMAL; *p* < 0.001 for both; SOY vs. SOY + Cr; *p* = 0.023 also for both, Figure 5B). Despite these differences, the integrity of nuclear and mitochondrial DNAs was not affected by diet, neither in situ (Figure 6A) nor after perfusion (Figure 6B). Oxidative and genotoxic stress are molecular hallmarks of DXR toxicity [34,40,41]. 

After DXR perfusion, TBARS tended to increase as compared to the control (P_DXR_ = 0.058, Figure 5B), but the lower TBARS and FRAP values in the SOY group as compared to NORMAL were preserved (Figure 5B). Consistent with our previous study in ex vivo Langendorff perfused heart [34], DXR caused extensive mitochondrial and nuclear DNA damage, but again, the extent of damage was unaffected by the three diet regimens (Figure 6B).

## 4. Discussion

This study reveals nutrition-induced alterations in cardiac function, cell signaling, and some biochemical markers after only 4 weeks of differential feeding of young male rats. The Cr-free soy-based diet (SOY) as compared to standard rodent diet (NORMAL) activated AMPK signaling as revealed by increased ACC phosphorylation, slightly increased the ADP/ATP ratio, and lowered both lipid peroxidation and the total antioxidant capacity. Supplementation of SOY with 2% Cr (SOY + Cr) as compared to SOY moderately increased cellular Cr, predominantly affected signaling pathways by activating Akt and ERK, and slightly increased cardiac developed pressure and contractility (LVDP and dp/dt) at baseline. These alterations are, in principle, relevant for cardiac health and its response to DXR, but they did not alleviate cardiac dysfunction induced by acute DXR challenge in the perfused heart model applied here.

Three key signaling pathways with fundamental importance for cardiovascular health were altered by diet: AMPK, ERK, and Akt. This may be critical to many functional and biochemical changes detected in our study. AMPK is a central energy sensor and regulator of the cell. During energy stress, it is activated allosterically by AMP and ADP, favors catabolism, and maintains cellular energy homeostasis [42]. Soy-based diet (SOY) as compared to standard chow (NORMAL) led to strong phosphorylation of ACC, an AMPK substrate, reporting activation of this pathway in the heart, consistent with a slightly reduced energy state in situ. Perfusion with DXR is known to induce a drop in the cardiac energy state [39], but paradoxically, this often occurs without activation of AMPK signaling, as reported by us [31,34] and others [43]. In the present study, we also observed signs of bioenergetic impairment by DXR in perfused heart, together with decreased AMPK activation. Only the SOY group maintained AMPK energy signaling as seen at the level of P-AMPK and P-ACC. Indeed, some treatments known to activate AMPK were shown to mitigate DXR cardiotoxic effects, including diet restriction [43,44]. Different soy components were implicated in AMPK activation in tissues other than heart, such as phytoestrogens in rat [45], genistein in cultured cancer cells [46], and different types of polyphenols [47,48]. The addition of Cr (SOY + Cr) did not (further) activate cardiac AMPK, consistent with a study on skeletal muscle [49], and not supporting earlier data on muscle cells [50].

Phosphorylation and activation of the rather pro-anabolic Akt and ERK by diet in heart in situ occurred rather inversely relative to AMPK. While the soy diet (SOY vs. NORMAL) increased AMPK activity and left Akt activity unchanged or tended to diminish ERK activity, the addition of Cr (SOY + Cr vs. SOY) led to activation of Akt and ERK, with a trend of lower AMPK activity. This supports a negative cross-talk of these kinases as described by us [34] and others [34,51,52,53], and by which AMPK is inhibited via Akt-dependent phosphorylation in its catalytic α-subunit. This cross-talk can modulate AMPK activity in the heart both under basal conditions in situ and during DXR perfusion [34]. The inhibitory effects of the soy-based diet on Akt and ERK could be mediated by genistein, known to inhibit Tyr kinases (for a review, see [54]) and to have an anti-proliferative effect, consistent with indirect AMPK activation [55,56,57,58,59]. The activation of Akt and ERK seen with Cr supplementation was also reported for skeletal muscle [60,61] and suggested by recent database meta-analysis [62]. Such rather pro-anabolic effects could mediate many cytoprotective aspects of Cr supplementation, such as in cardioprotection [18,19], muscular dystrophies, neuromuscular and neurodegenerative disorders [16,63], brain health [64], or wound healing [65]. Notably, upregulation of Akt was shown to confer significant cardioprotection in DXR-treated animals [66].

Slightly altered performance of the perfused heart was observed only after Cr supplementation (SOY + Cr vs. SOY) under baseline conditions. Contractility (dp/dt) and developed pressure (LVDP) were modestly increased, together with a trend of a decreased heart rate, with the latter resulting in an unchanged rate pressure product (RPP). Beyond bioenergetics, Cr enhances the expression of muscle myogenic regulatory factors as reported for skeletal muscle [60,61,67,68] and affects signaling pathways, such as the Akt activation mentioned above. An earlier study did not detect Cr-induced changes in cardiac function [69], possibly because the reference diet plays an important role. Soy is not only Cr-free but may itself have additional cardio-vascular effects not examined here, such as blood pressure-lowering effects [70,71,72,73,74]. Moreover, the higher basal activity of the AMPK pathway may potentiate Cr effects, since both are directed to improve cell energetics. Perfusion with DXR deteriorated cardiac function as expected, but diet did not modulate the functional response, except for a lower increase in diastolic pressure in the SOY + Cr vs. SOY group. Cr was also not effective in a perfused heart model for acute oxidative stress [75]. Possibly, the acute insult at a supraclinical DXR dose is too strong, and long-term exposure of animals to low clinical DXR doses would be more suitable for an analysis of dietary effects.

A striking feature of the soy-based diet (SOY vs. NORMAL) was the low cardiac level of both lipid peroxidation and total antioxidant capacity. This was most pronounced in the ex vivo perfused heart, likely because of perfusion-associated oxidative stress. The antioxidant properties of soy include the main soy isoflavone genistein [76] and other phytoestrogens or polyphenolic compounds. They share a high reactivity as hydrogen or electron donors, can stabilize unpaired electrons as polyphenol radicals, chelate transition metal ions, modulate the expression of antioxidant defense genes, and activate signaling pathways [77,78]. However, a general lipid-lowering capacity of soy could also reduce detectable lipid peroxides [27,54,79], consistent with AMPK-induced reduction of lipid anabolism and an increase of their catabolism. The combined reduction of both lipid peroxidation and total antioxidant capacity may seem surprising, but the latter is likely an adaptation to the lower oxidative stress levels in the SOY group, as also indicated by literature data [80]. Cr supplementation had no such dramatic effects. Further, diet-related differences in oxidative stress were not reflected in oxidative DNA lesions, because these were either below the detection threshold, or they were rapidly removed by repair systems. Thus, mt and nDNA are unlikely targets and/or mediators of the diet-related effects described herein.

Regarding Cr, it should be emphasized that supplementation can only modestly increase cardiac Cr levels, as observed here and in earlier studies [69]. With increasing cellular Cr, a feedback mechanism downregulates the creatine transporter in charge of cellular Cr uptake [69]. Nevertheless, even this moderate increase in intracellular Cr was sufficient to trigger some significant cardiac effects.

Finally, our study calls for a note of caution with respect to diets used in animal studies. Already basic formulations likely contain ingredients with considerable biological activity. In particular, the soy-derived products commonly used in rodent chow (soy meal or protein isolate) must be considered as bioactive agents or even nutraceuticals [28]. The present study used a soy-based diet as a genuine Cr-free control chow for Cr supplementation studies. However, replacing 4% fish hydrolysate with 4% soy protein isolate already generates a bio-active diet. Soy-derived products contain isoflavones (genistein, daidzein, and equol) that are qualified as phytoestrogens due to their ability to act in the body as estrogens or selective estrogen receptor modulators [72]. Thus, caution is advised when translating results obtained with animals fed a high-soy diet directly to humans, especially those consuming a traditional Western diet. The quantity of circulating phytoestrogens in rats ingesting a soy-based diet may be comparable to Asian people who eat a soy-based diet [28]. Even if soy dietary supplements became popular in vegetarian/vegan cuisine, the overall benefit and/or safety of this diet is still a matter of debate (for a review, see [27,28,29,30]). Obviously, phytoestrogens may negatively affect the reproduction system, mainly in males and children [28]. In view of these controversies, the controlled use of soy or other bioactive compounds in animal diets and the explicit analysis of their effects is highly desirable. In this context, the choice of animal gender should also be considered. For example, practically all animal studies on DXR cardiotoxicity were conducted with male animals and these are then compared to human studies performed with both genders, although gender may affect the cardiac response to DXR. Earlier literature suggested that female sex is a risk factor for DXR cardiotoxicity [81,82,83], but most recent reports show that female sex hormones may protect against DXR cardiotoxicity by reducing oxidative stress and proinflammatory responses [84,85]. One may even ask whether phytoestrogens could successfully mimic this effect.

## 5. Conclusions

In conclusion, a soy-based diet alone or supplemented with Cr, fed for four weeks to rats, is sufficient to alter cardiac function, cell signaling, and biochemical markers of the energy state and oxidative stress. These effects are relevant for cardiovascular health but were not sufficient to alleviate cardiac dysfunction induced by a supraclinical DXR concentration in the perfused rat heart model. However, whether these diets could affect the long-term response to chronic and clinically relevant DXR doses in the rat model described here, or in human patients treated with DXR, remains to be established.

## Data Availability

Data supporting the findings of this manuscript are available from the corresponding authors upon reasonable request.

## Supplementary Materials

The following are available online at https://www.mdpi.com/article/10.3390/nu14030583/s1, Figure S1: Time course of DXR effect on heart function.
