# Supplementary material for: Supplementing Soy-Based Diet with Creatine in Rats: Implications for Cardiac Cell Signaling and Response to Doxorubicin
Source: Nutrients. 2022 Jan 28;14(3):583. doi: 10.3390/nu14030583 (PMC8840593; doi:10.3390/nu14030583)
Supplement: Supplementary file 1 [file nutrients-14-00583-s001.zip › nutrients-1539665-supplementary.pdf]

nutrients

MDPI

Article

Supplementing Soy-Based Diet with Creatine in Rats: Implications for Cardiac Cell Signaling and Response to Doxorubicin

Laurence Kay [1], Lucia Potenza [2], Isabelle Hininger-Favier [1], Hubert Roth [1], Stéphane Attia [1], Cindy Tellier [1], Christian Zuppinger [3], Cinzia Calcabrini [2], Piero Sestili [2], Theo Wallimann [4], Uwe Schlattner [1,*] and Malgorzata Tokarska-Schlattner [1,*]

Supplementary Figure S1

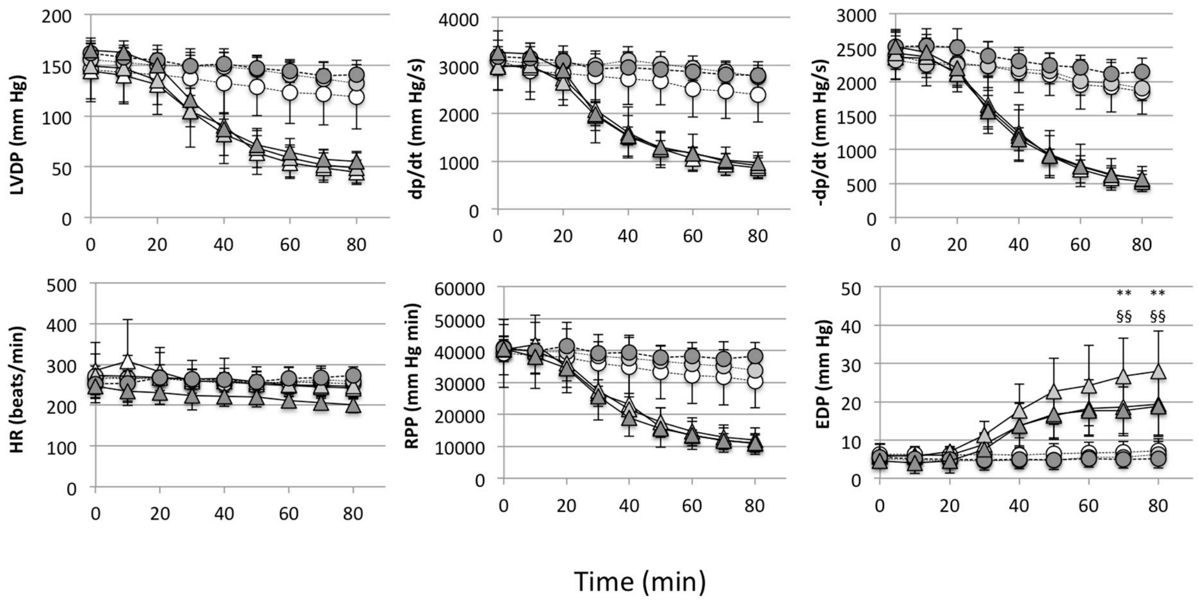

Figure S1. Time course of DXR effect on heart function. Hemodynamic parameters: left venticular developed pressure (LVDP), dp/dt, -dp/dt, heart rate (HR), rate pressure product (RPP) and end-diastolic pressure (EDP) measured in isolated rat hearts during 80 min perfusion without or with 25 µM DXR (circles or triangles respectively). Hearts were isolated from animals from different diet groups: NORMAL (white symbols), SOY (light grey symbols), SOY+Cr (dark grey symbols). In DXR perfused hearts, statistically significant differences between diet groups are marked: ** $P < 0.01$ SOY *vs.* SOY+CR, §§ $P < 0.01$ SOY *vs.* NORMAL ($n \geq 4$). Statistics: Time-courses for functional parameters in perfused isolated hearts were analyzed using mixed models, with diet and perfusion as between groups fixed effects, time as within group repeated effect and subject as random effect. Interactions were tested and post hoc analysis was realized, when appropriate, with Bonferroni correction for multiple testing (Stata 13; Stata Corp., College Station, TX, USA).
